# The effect of acupuncture on tumor growth and gut microbiota in mice inoculated with osteosarcoma cells

**DOI:** 10.1186/s13020-020-00315-z

**Published:** 2020-04-07

**Authors:** Xiaoru Xu, Xiangru Feng, Min He, Zepeng Zhang, Jiajia Wang, Haiyu Zhu, Tie Li, Fuchun Wang, Mengmeng Sun, Zhihong Wang

**Affiliations:** 1 Changchun University of Chinese Medicine, No. 1035, Boshuo Rd, Jingyue Economic Development District, Changchun, 130117 China; 2 Key Laboratory of Polymer Ecomaterials, Changchun Institute of Applied Chemistry, Chinese Academy of Sciences, 5625 Renmin Street, Changchun, 130022 People’s Republic of China; 3 Research Center of Traditional Chinese Medicine, The Affiliated Hospital to Changchun University of Chinese Medicine, Changchun, Jilin China; 4 SKL of Quality Research in Chinese Medicine, Institute of Chinese Medical Sciences, University of Macau, N22 Avenida da Universidade, Taipa, Macau China

**Keywords:** Acupuncture, Gut microbiota, Xenograft, Osteosarcoma

## Abstract

**Background:**

Cancer is a complex systemic disease. As a key component of traditional Chinese medicine, acupuncture is a clinically proven medical treatment for many diseases, and it also has preventative effects as it balances the body, allowing it to self-regulate. For cancer patients, acupuncture is widely used as complementary therapy to boost the immune system and reduce the side effects of radiotherapy and chemotherapy. However, few studies have determined how acupuncture against cancer, especially in regulating the intestinal flora of the tumor-burdened mice.

**Methods:**

We treated osteosarcoma tumor-burdened mice by using needling on different acupoints and acupoints combination, thereafter determined the effects of acupuncture on tumor growth by using imaging technology in vitro. In addition, intestinal bacteria were analyzed for further understanding the holistic and systemic treatment effects of acupuncture in osteosarcoma tumor-burdened mice.

**Results:**

Acupuncture treatment can delay tumor growth and changes of intestinal bacteria in osteosarcoma tumor-burdened mice. In detail, the loss of body weight and the development of tumor volume of mice have been postposed by needling specific acupoints. In addition, acupuncture treatment has delayed the changes of the relative abundance of *Bacteroidetes*, *Firmicutes* and *Candidatus Saccharibacteria* at the phylum level. Moreover, the relative abundance of many bacteria (e.g., *Catabacter*, *Acetatifactor* and *Aestuariispira*) has been regulated by using acupuncture treatment, and the trend of structural changes of these bacteria at the genus level has also been postposed compared to that of the tumor-burdened mice model group.

**Conclusion:**

Our results suggest that acupuncture may provide a systemic treatment for cancer. Our findings encourage new and extensive research into the effects of acupuncture on changes of the intestinal microbiome associated with the development of cancer.

## Background

Cancer is a major public health problem worldwide and is a leading cause of death in both more and less economically developed countries [1]. Accordingly, most recent estimates demonstrate that all countries face huge challenges in managing the very large and increasing burden of cancer, which will only increase in the future [2]. Therefore, the investigation of cancer has become a hot area in scientific research, and various novel biomedical technologies (e.g., genomics, proteomics, and metabolomics) have been used in this area [3–8]. Recently, increasing studies have demonstrated that the gut microbiota plays an important role in regulating of human health and disease by maintaining gut homeostasis [9, 10]. Dysfunction of gut microbiota is associated with many diseases, including gastrointestinal diseases [11–14] and cancer [15, 16]. Microorganisms have important effects on cancer; for example, *Helicobacter pylori*, *hepatitis B and C viruses*, and *human papillomavirus* have been recognized to cause cancer [17–20]. In addition, an increasing number of comparative studies have shown that there are differences in gut microbiota composition between cancer patients and healthy individuals, and the occurrence of cancer often causes or accompanies changes of the intestinal flora [21–23]. Those reported cancers with changes in intestinal flora include not only gastrointestinal cancers (e.g., stomach and colorectal cancer) [15, 16], but other cancer types as well (e.g., pancreas, liver, prostate, and breast cancer) [24–29]. As research provides evidence and expands our understanding of carcinogenic mechanisms, studies on intestinal flora present many more opportunities to develop therapies for cancer diagnosis and management.

Meanwhile, given the widespread cases of cancer worldwide, especially in China [1], an increasing number of complementary and alternative therapies are used in cancer treatment and management. Acupuncture is an important complementary therapy based on the theories and principles of Chinese medicine, it has wide applications and is safe, economical, and convenient with few side effects [30]. To date, acupuncture therapy has been widely used to treat various diseases, such as pain [31], chronic obstructive pulmonary disease [30], breast cancer [32], asthma [33], allergic rhinitis [34], and mild cognitive impairment [35]. The use of acupuncture to treat a variety of conditions associated with disease has attracted the attention of scientists worldwide. In tradition, acupuncture is used to treat diseases caused by blockages of “Qi” and “Xue” [36], which leads to good therapeutic effect of acupuncture on pain [31], including cancer pain [37]. The physiological basis of the analgesic action of acupuncture has been investigated using modern science and technology, and the analgesic effects of acupuncture may attribute to the modulation of the 5-hydroxytryptamine signalling system, the adrenergic system and the *N*-methyl-d-aspartic acid/AMPA/kainate signalling system etc. [31]. In addition, acupuncture is a comprehensive and holistic treatment. With the development of omics technology, the therapeutic effect of acupuncture has been studied in more diseases (e.g., diseases of digestive system), and some protein and metabolism markers have been found out to indicate the effect of acupuncture by using proteomics and metabolomics [38, 39]. Moreover, many research centers in Europe and the United States have tried to integrate acupuncture with conventional cancer treatment [40, 41]. Currently, the use of acupuncture in cancer has focused on improving the clinical symptoms of cancer patients and reducing the side effects caused by radiotherapy and chemotherapy [42–44]. A few studies have focused on the apoptosis of cancer cells and expression of gene and protein (e.g., CyclinD1 and CDK4) in order to indicate the therapeutic effect of acupuncture on tumor model animals [45–47]. However, research on whether acupuncture can delay the growth and metastasis of tumors in vivo is limited, and the biomedical evidence for acupuncture treatment of cancer also needs to be further explored. Recently, some studies have linked the effects of acupuncture with changes in intestinal flora to study the regulatory effects of acupuncture (or moxibustion) on obesity and ulcerative colitis [48–50]. These studies have suggested that gut microbiota may be a novel target for the effects of acupuncture treatment. Therefore, study of the regulation of intestinal flora combined with treatment effects of acupuncture on cancer in vivo may provide a new view for understanding the therapeutic effect of acupuncture on cancer.

In this research, we treated osteosarcoma tumor-burdened mice by using acupuncture, and determined the effects of acupuncture on tumor growth by using imaging technology in vitro. In addition, the intestinal bacteria were analyzed for further understanding the holistic and systemic treatment effects of acupuncture in osteosarcoma tumor-burdened mice. The results showed acupuncture treatment can delay tumor growth and changes of intestinal bacteria in osteosarcoma tumor-burdened mice, thereby providing new insights into the investigation of acupuncture treatment.

## Materials and methods

### Animals

Female BALB/c mice (16–18 g; aged 6–8 weeks) were managed and housed in the Experimental Animal Center of Changchun University of Chinese Medicine (Jilin, China). The protocols were approved by the Ethics Committee of the Changchun University of Chinese Medicine (No. 20180107).

### Cell culture and establishment of a mouse model of osteosarcoma

**Fig. 1:**
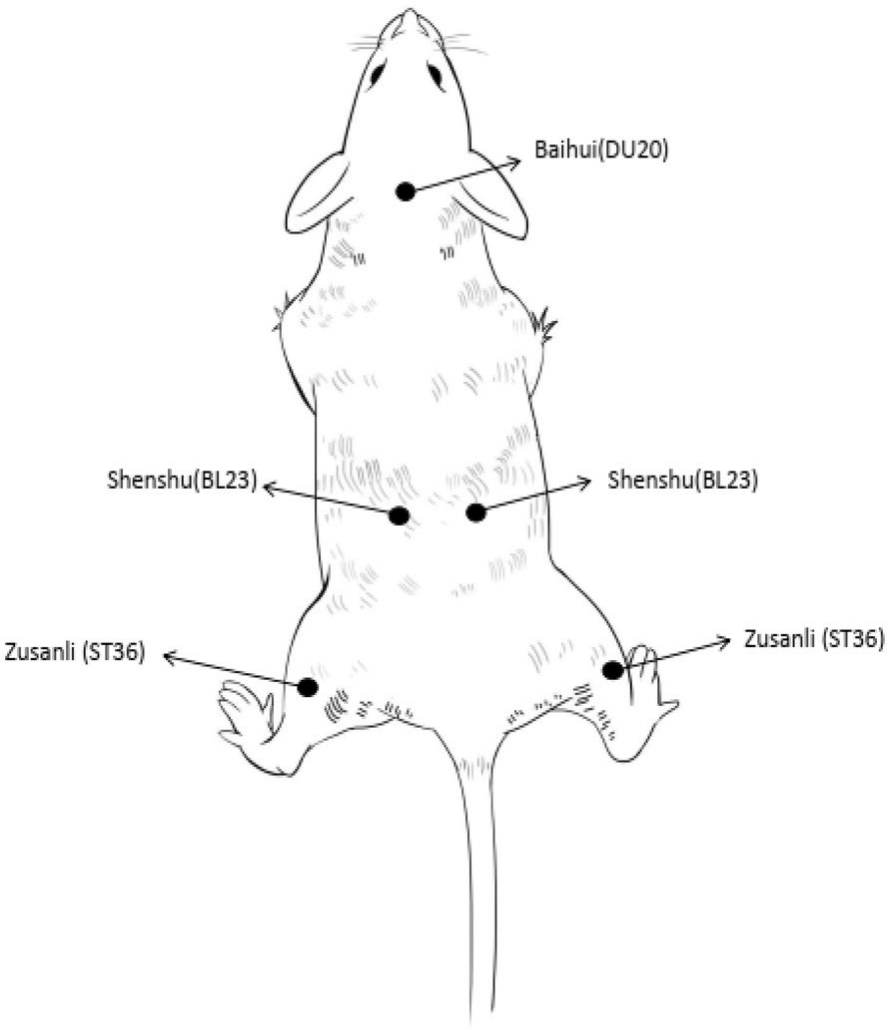
Schematic diagram of acupuncture points on the body of a mouse. Five acupoints were selected for acupuncture treatment. BL23 and ST36 were symmetrical on both sides of the body

The mouse osteosarcoma K7 cell line was maintained in complete Dulbecco’s Modified Eagle’s Medium (Hyclone, South Logan, UT, USA) supplemented with 10% fetal bovine serum (FBS; Hyclone) at 37 °C. Cells were harvested after reaching near confluency. Experiments were not continued if cell viability was < 90%, as assessed by trypan blue staining. Control (n = 10, non-tumor vaccination), model (n = 10, non-acupuncture treatment), and acupuncture treatment (n = 10, specific acupoints groups and acupoints combination group, each group has ten animals) groups were established. Briefly, mice in the model and treatment groups were injected hypodermically with K7 cells (3 × 10^6^ cells in 100 μL saline), while the control group was hypodermically injected with saline alone. Acupuncture of mice in the treatment group was initiated when the tumor volume reached approximately 70 mm^3^. The whole treatment process was divided into three phases: phase I (from days 0 to 5, total of 6 days) was the baseline phase, which was the period after tumor implantation and before treatment; phase II (from days 6 to 12, total of 7 days); and phase III (from days 14 to 20, total of 7 days) was the acupuncture treatment period, with no treatment on day 13. Acupuncture at Shenshu (BL23), Baihui (DU20), and Zusanli (ST36) acupoints (Fig. 1) was performed once a day for 2 weeks. The short and long diameters of the subcutaneous osteosarcoma xenograft and the weight of the mice were measured every 2 days for 2 weeks. Measure tumor short diameter and long diameter (mm) with vernier caliper every 2 days for 2 weeks. The tumor volume was calculated by the following equation: (short diameter)^2^ × (long diameter)/2.

### Tumor imaging

The d-luciferin potassium salt was purchased from Solarbio (D8930; Beijing Solarbio Science & Technology Limited Company, Beijing, China). The absorption time of the drug is 10–15 min, and radioactivity and cell survival cannot be detected. Before observation, 15 mg/mL luciferin potassium solution was injected into each mouse intraperitoneally at a concentration of 10 mg/g body mass. After 5 min, the mice were injected with 1% pentobarbital sodium (50 mg/kg) intraperitoneally according to body weight, and tumor growth was observed by in vivo imaging using the In-Vivo Xtreme system (Xtreme BUX00081; Bruker Corporation, Billerica, MA, USA).

### Flow cytometry assay

Ex vivo analysis of different groups of T cells was done using a flow cytometer (BD FACS Aria II; BD Biosciences, San Jose, CA, USA). To study the immune cells in the spleen and lymph, tissues were harvested from mice in different groups and stained with anti-CD3-cy5.5 (Catalog: 100326; BioLegend, San Diego, CA, USA), anti-CD8a-APC (Catalog: 100712; BioLegend), and anti-CD4-PE (Catalog: 100408; BioLegend) antibodies according to the manufacturer’s protocols. Briefly, tissues were cut into small pieces and put into a glass homogenizer containing phosphate-buffered saline (pH 7.4) with 2% heat-inactivated FBS. Then a single-cell suspension was prepared by gentle pressure with the homogenizer without addition of digestive enzymes. Finally, cells were stained with fluorescence-labeled antibodies after the removal of red blood cells (RBCs) using RBC lysis buffer. Anti-CD45-FITC was used to identify the T cells. Based on these groups, cytotoxic T lymphocytes (CTLs) and helper T cells were CD3+CD4−CD8+ and CD3+CD4+CD8−, respectively. To analyze the NK T cells, cells in the lymph were stained with anti-CD45-FITC (Catalog: 103108; BioLegend), anti-CD3-cy5.5 (Catalog: 100326; BioLegend), and anti-49b-APC (Catalog: 103516; BioLegend) antibodies according to standard protocols as CD45+CD3−CD49b+. All of the reagents and flow cytometers used were from Biolegend (BD Biosciences). A single-cell suspension of lymph nodes was prepared using the same protocol as that for spleen tissues. All of the antibodies used in our experiments were diluted 200-fold. All gate strategy and FACS data of different staining schemes were presented in Additional file 1: Original data 1, Additional file 2: Original data 2.

### Enzyme-linked immunosorbent assay

Main chemicals and instruments: Multiskan Spectrum (SpectroNano S/N 601-1175; BMG LABTECH, Offenburg, Germany), a mouse tumor necrosis factor-alpha (TNF-α) enzyme-linked immunoassay (ELISA) kit (PT512), Mouse interferon gamma (IFN-γ) ELISA kit (PI508), Mouse interleukin 6 (IL-6) ELISA Kit (PI326), and BCA Protein Assay Kit (P0012) were purchased from Beyotime (Shanghai BiYunTian Biotechnology Co. Ltd., Shanghai China). The spleen tissue of mice from three groups was ground after treatment, followed by ELISA to measure the levels of IFN-γ. Blood was collected from the eyeball of the mice and transferred to an anticoagulant tube. After incubation at room temperature for 2 h, the blood was centrifuged at 1000*g* for 20 min, and the ELISA was performed using the supernatant (serum) to measure the levels of TNF-α and IL-6 in the serum.

### Sample processing and DNA extraction from stool

Fecal samples were collected in the morning and stored at − 80 °C until DNA extraction. Microbial DNA was extracted from the fecal samples with the QIAamp^®^ DNA Stool Mini Kit (Qiagen, Hilden, Germany), according to the manufacturer’s protocol. After quantification, PCR amplification of the V3–V4 region of the microbial 16S rRNA gene was performed with primers (forward: 5-CCTACGGGNGGCWGCAG-3, reverse: 5-GACTACHVGGGTATCTAATCC-3). After electrophoresis, the PCR products were purified on a 1.2% agarose gel with the QIAquick Gel Extraction Kit (Qiagen). Libraries were sequenced by Genesky Biotechnology, Inc. (Shanghai, China) using an MiSeq Benchtop Sequencer.

### Bioinformatics analysis

Bioinformatics analysis was performed with the help of Genesky Biotechnologies, Inc. The raw sequences were sorted into different samples by matching the barcodes. Subsequently, the sequences of primers and barcodes were trimmed using CUTADAPT (cutadapt.readthedocs.io/en/stable/#). The processed reads were merged using FLASH [34] and analyzed with the UPARSE [51] pipeline to generate an operational taxonomic unit (OTU) table. MOTHUR was used to convert the OTU table into a suitable input file for further alpha and beta analysis [52, 53]. The relative levels of OTU richness across all of the compost samples were compared by a rarefaction curve. To compare variations among the different samples, weighted UniFrac distances were calculated for principal coordinate analysis (PCoA) [54, 55]. Linear discriminant analysis (LDA) Effect Size (LEfSe), an algorithm for high-dimensional biomarker discovery, was used to identify the dominant factors responsible for the differences among three group [56]. Finally, a bacterial community heatmap was visualized using R package pheatmap.

### Statistics

Statistical analysis was performed using SPSS 23.0. One-way analysis of variance (ANOVA) and Student’s *t*-test were conducted for comparisons among the multiple groups. *p* < 0.05 was considered to be statistically significant.

## Results

**Fig. 2:**
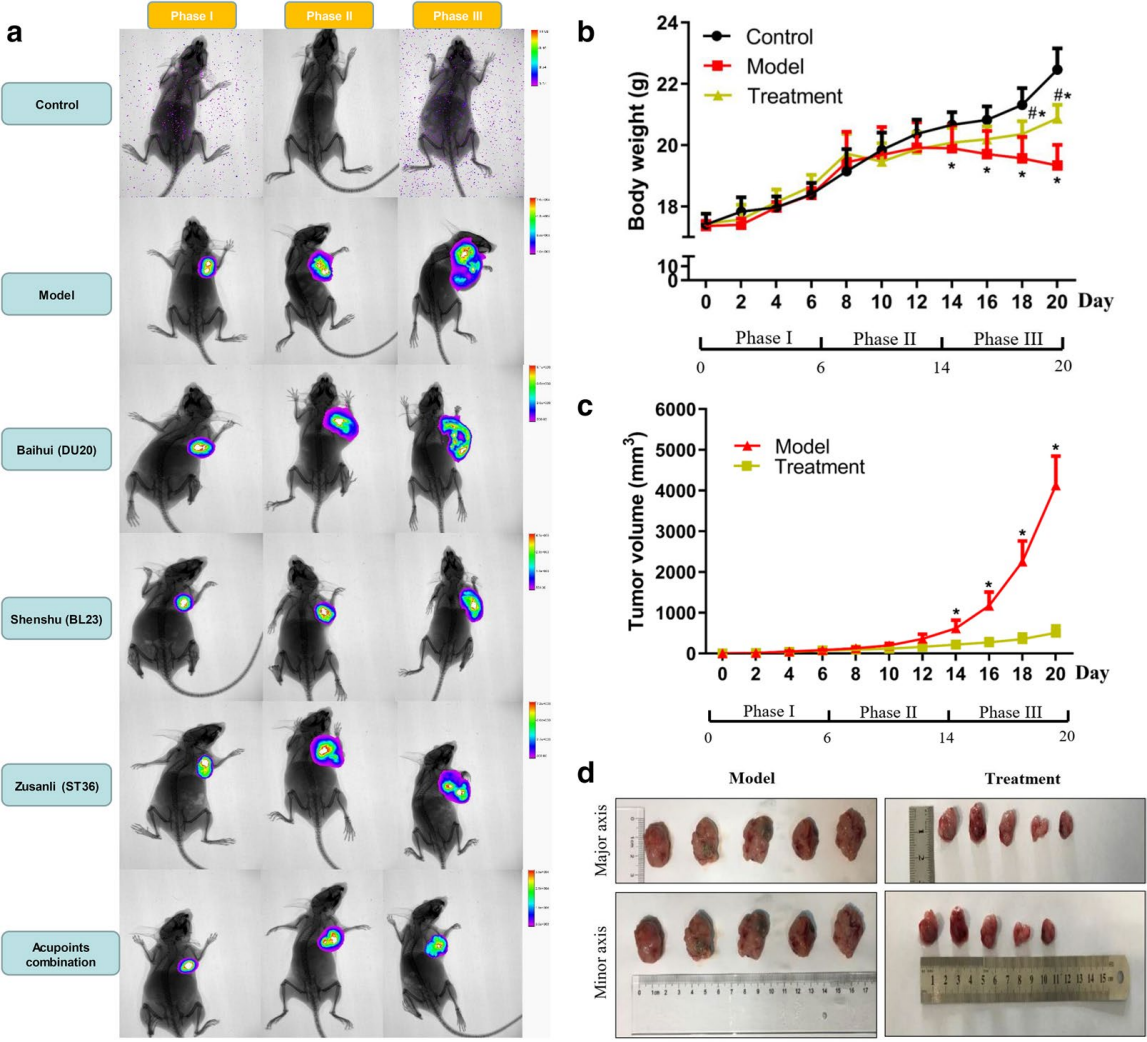
Body weight and tumor size of the mice. **a** Bioluminescence image of mice by using acupuncture treatment. **b** The body weight of control, model and acupoints combination treatment groups. *Indicates model or treatment group versus control group, *p* < 0.05 (two-tailed, unpaired Student’s *t*-test). ^#^Indicates model group versus treatment group, *p* < 0.05 (two-tailed, unpaired Student’s *t*-test). **c** Tumor volumes of mice in the model and treatment groups. *Indicates treatment group versus model group, *p* < 0.05 (two-tailed, unpaired Student’s *t*-test). **d** Schematic diagram of tumor size of mice in the model and treatment groups after the acupuncture treatment procedure

To observe the effects of acupuncture on subcutaneously transplanted osteosarcoma mice, we treated the mice using acupuncture at different acupoints and acupoints combination for 14 days (from phase II to phase III; Fig. 2b). In addition, in vivo imaging technology has been used to determine the tumor size in the end of each phase. Figure 2a indicates that the tumor size was relatively smaller in acupuncture treatment groups compared to that in model group, especially in the period of phase III, and needling acupoints combination may demonstrate best treatment effect in the images (Fig. 2a). To further evaluate the treatment effect between needling specific acupoints and acupoints combination, we measured tumor size and weight. The results showed that the tumor size and weight obtained by the treatment of needling acupoints combination were significantly lower than that of needling specific acupoints (Additional file 3: Fig. S1). Therefore, needling acupoints combination was used to conduct further studies and analysis in this research. Next, Fig. 2b shows that there was no significant difference in body weight of mice among the control, model and acupoints combination treatment groups in the first two phases. From days 14 to 20, the mice of model group had a significantly lower body weight than that of the control group (Fig. 2b). From days 18 to 20, both the mice of model and acupoints combination treatment groups had a significantly lower body weight than that of the control group, but the mice body weight of acupoints combination treatment group was significantly higher than that of model group (Fig. 2b). In addition, the tumor size continuously increased in the model group, but the increase of tumor size of acupoints combination treatment group was relatively slow (Fig. 2c). From days 14 to 20, the mice in model group had a significantly higher tumor volume than that in the acupoints combination treatment group (Fig. 2c, d). These results indicate that needling acupoints-especially acupoints combination-may against burdened tumor of mice. The effect of acupuncture on burdened tumor can be attribute to delay of tumor growth.

**Fig. 3:**
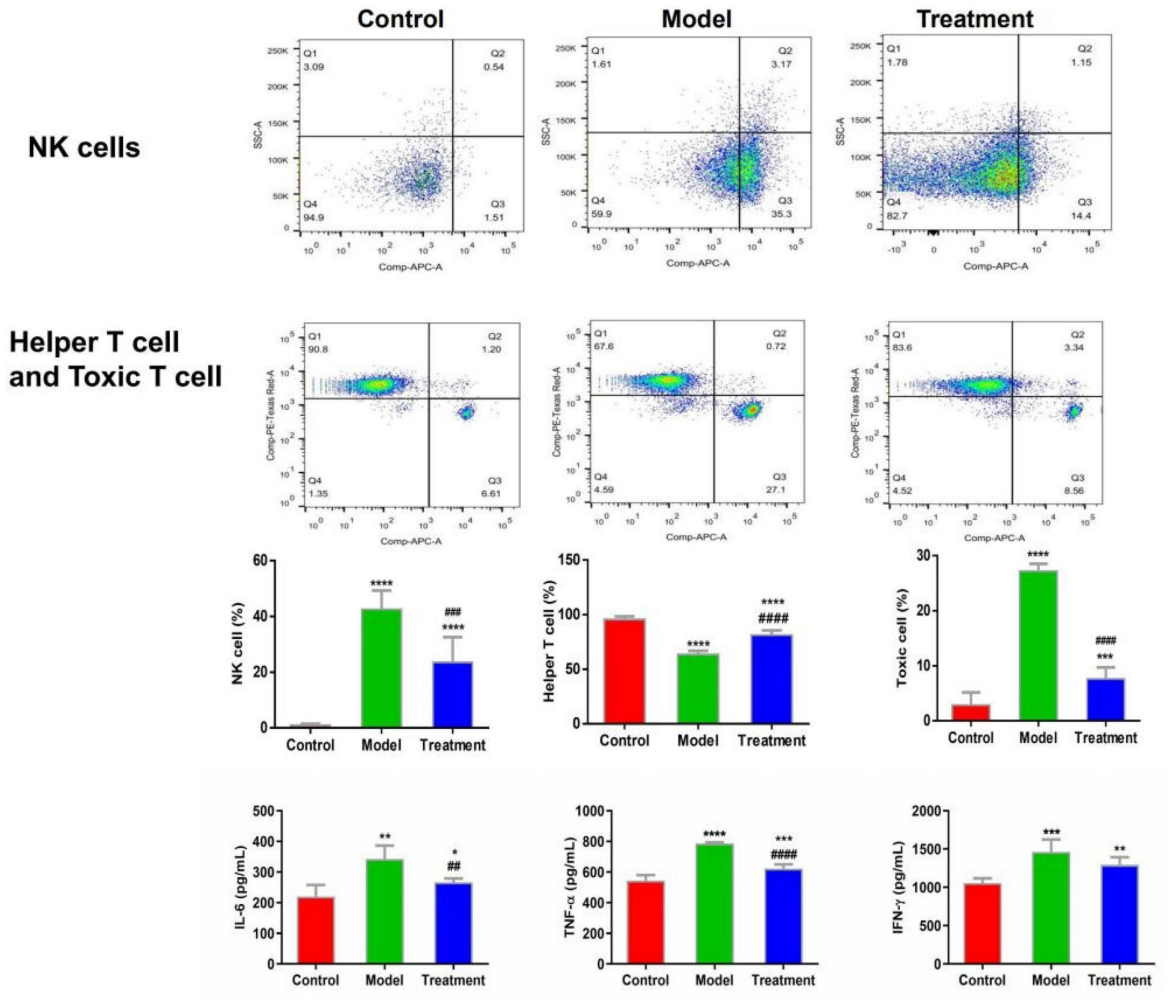
Flow cytometry results and levels of immune cells and inflammatory factors in the three groups. *Indicates model or treatment (acupoints combination) group versus control group. *p < 0.05; **p < 0.01; ***p < 0.001; ****p < 0.0001 (two-tailed, unpaired Student’s *t*-test). ^#^Indicates treatment group (acupoints combination) versus model group. ^##^*p *< 0.01; ^###^*p *< 0.001; ^####^*p *< 0.0001 (two-tailed, unpaired Student’s *t*-test)

**Fig. 4:**
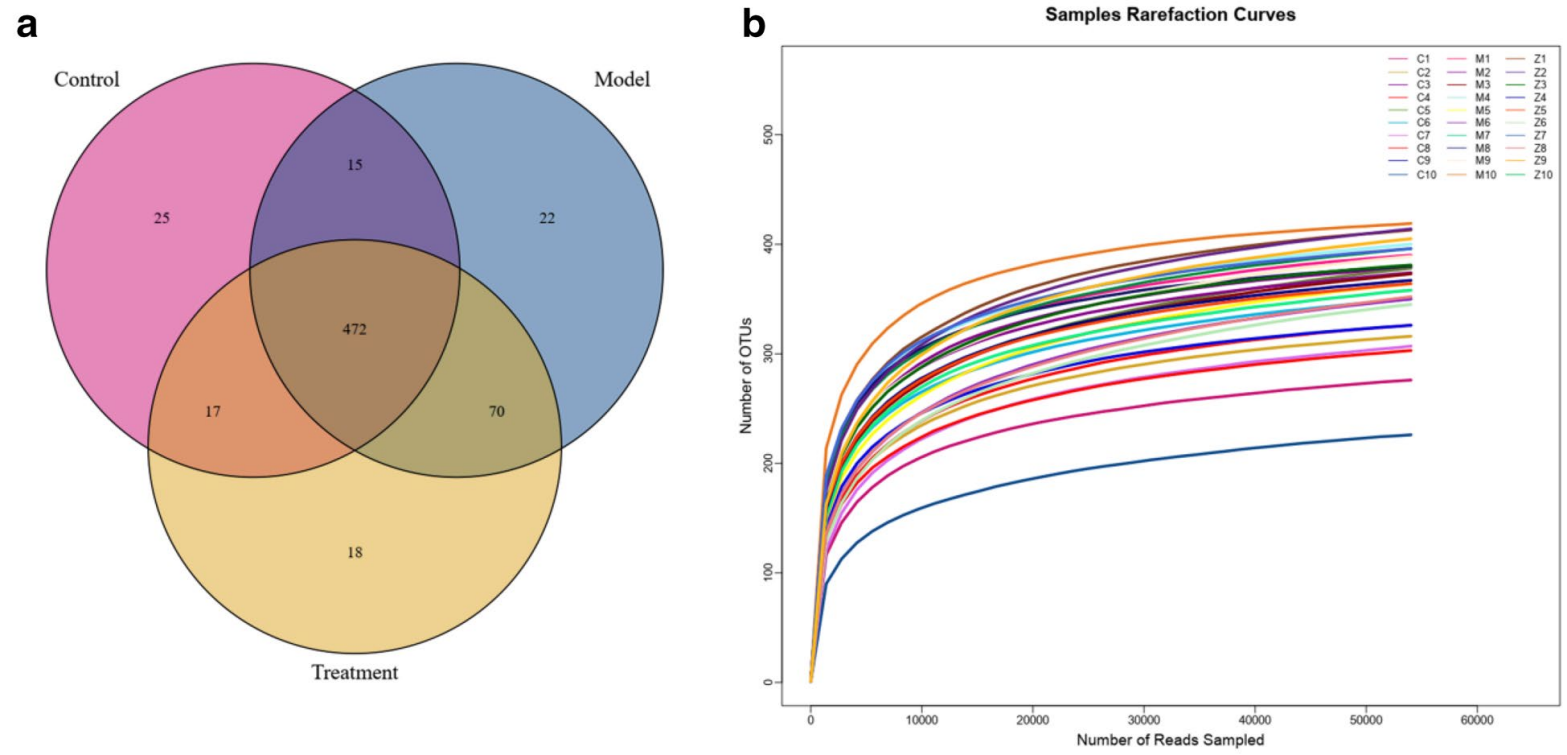
Evaluation of sequencing data from the mice fecal samples. **a** Number of species of bacterial taxa of mice in three groups; **b** rarefaction curves calculated from the flora sequencing data using Sobs index of OTU level. Treatment group indicates acupoints combination treatment group

**Fig. 5:**
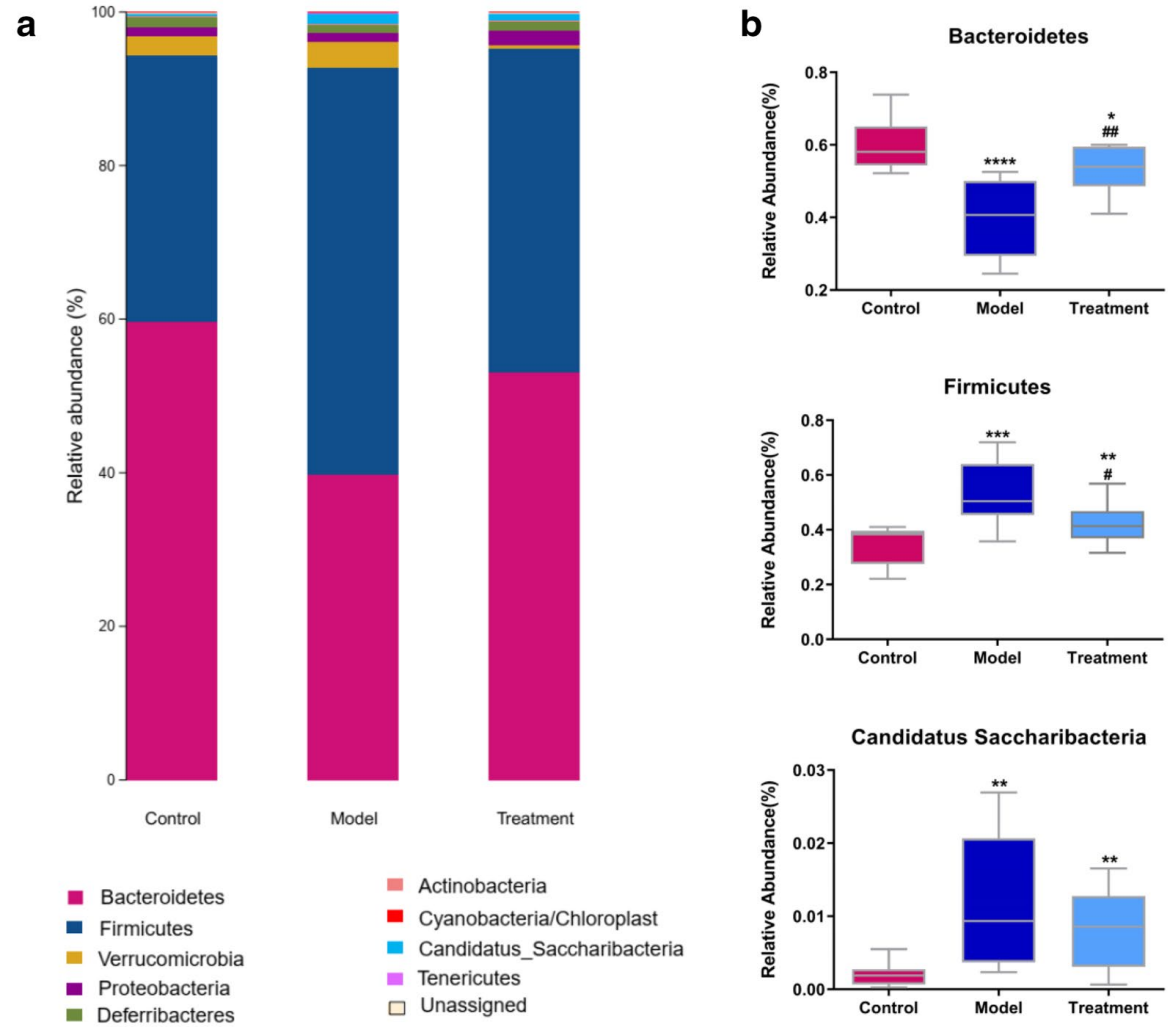
Microbial community structures at the phylum level of mice in the three groups. **a** Relative abundances (%) of bacterial taxa at the phylum level. **b** Significantly different bacterial taxa at the phylum level of mice in the three groups. *Indicates model or treatment (acupoints combination) group versus control group. *p < 0.05; **p < 0.01; ***p < 0.001; ****p < 0.0001 (one-way ANOVA). ^#^Indicates treatment (acupoints combination) group versus model group. ^#^*p* < 0.05; ^##^*p* < 0.01; (one-way ANOVA)

**Fig. 6:**
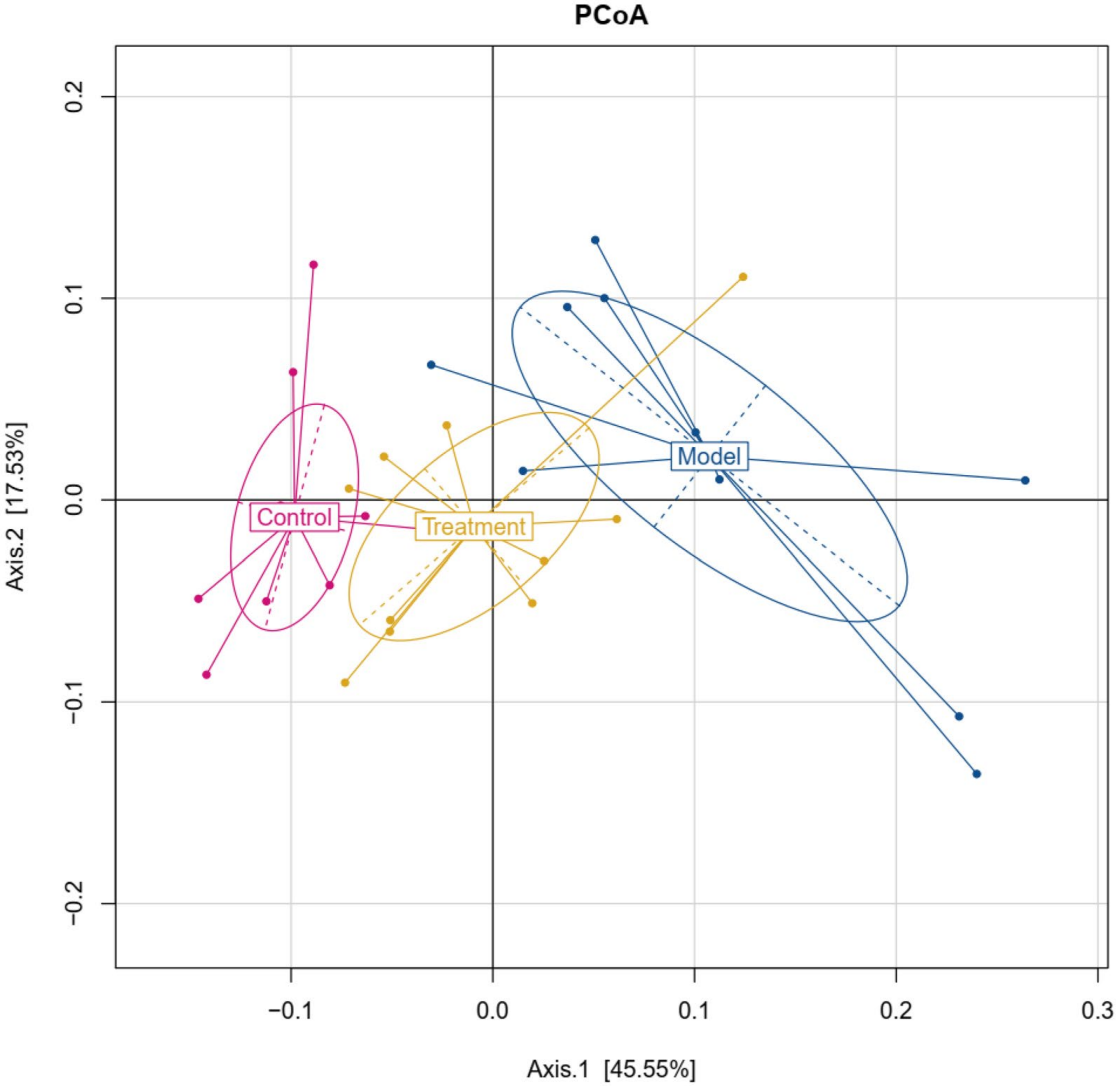
PCoA of beta diversity calculated on the unweighted UniFrac distances using OTU data at the phylum level of mice in the three groups. Treatment group indicates acupoints combination treatment group

**Fig. 7:**
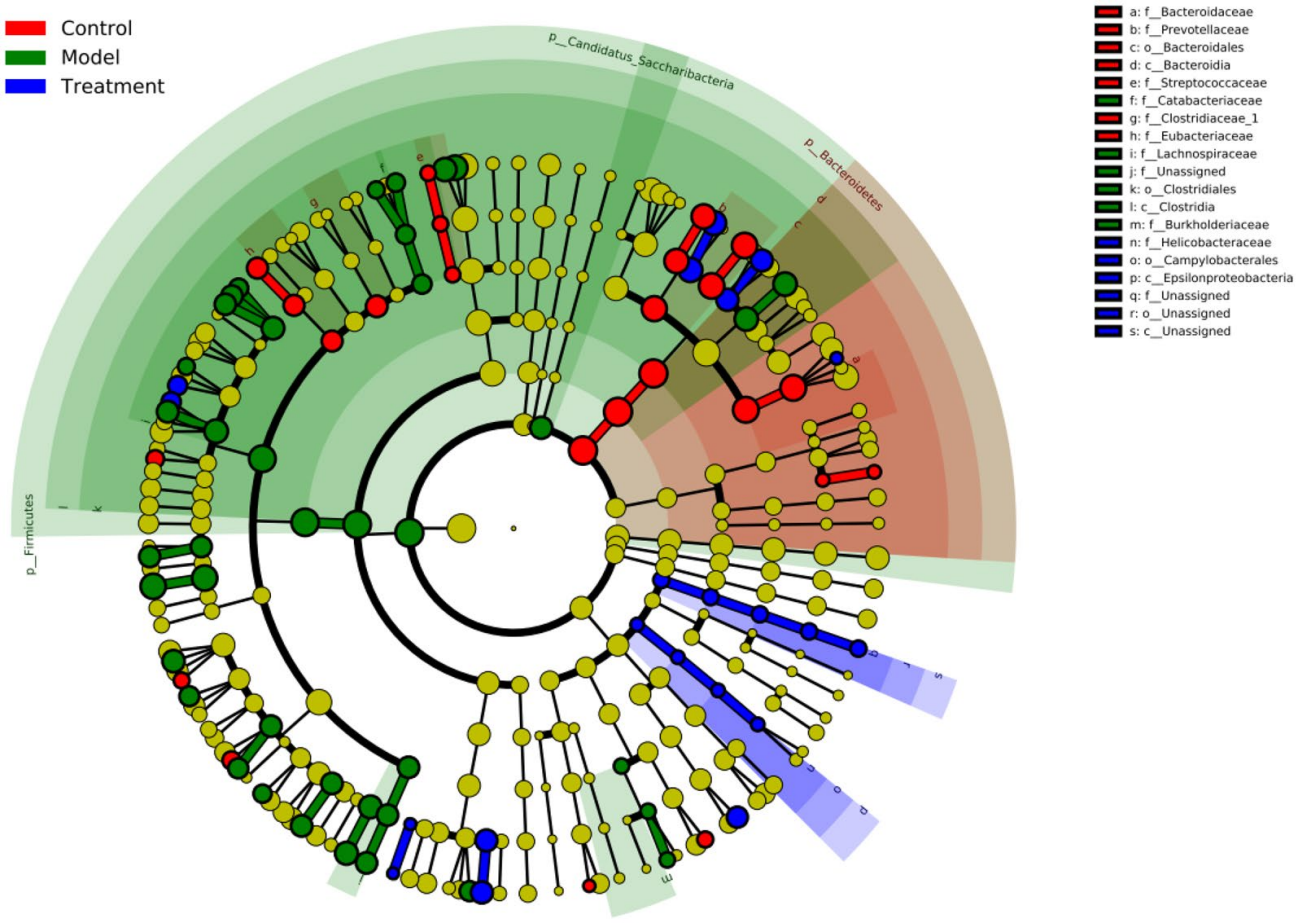
Cladograms generated by LEfSe indicating differences in the bacterial taxa of mice among the three groups. Treatment group indicates acupoints combination treatment group

**Fig. 8:**
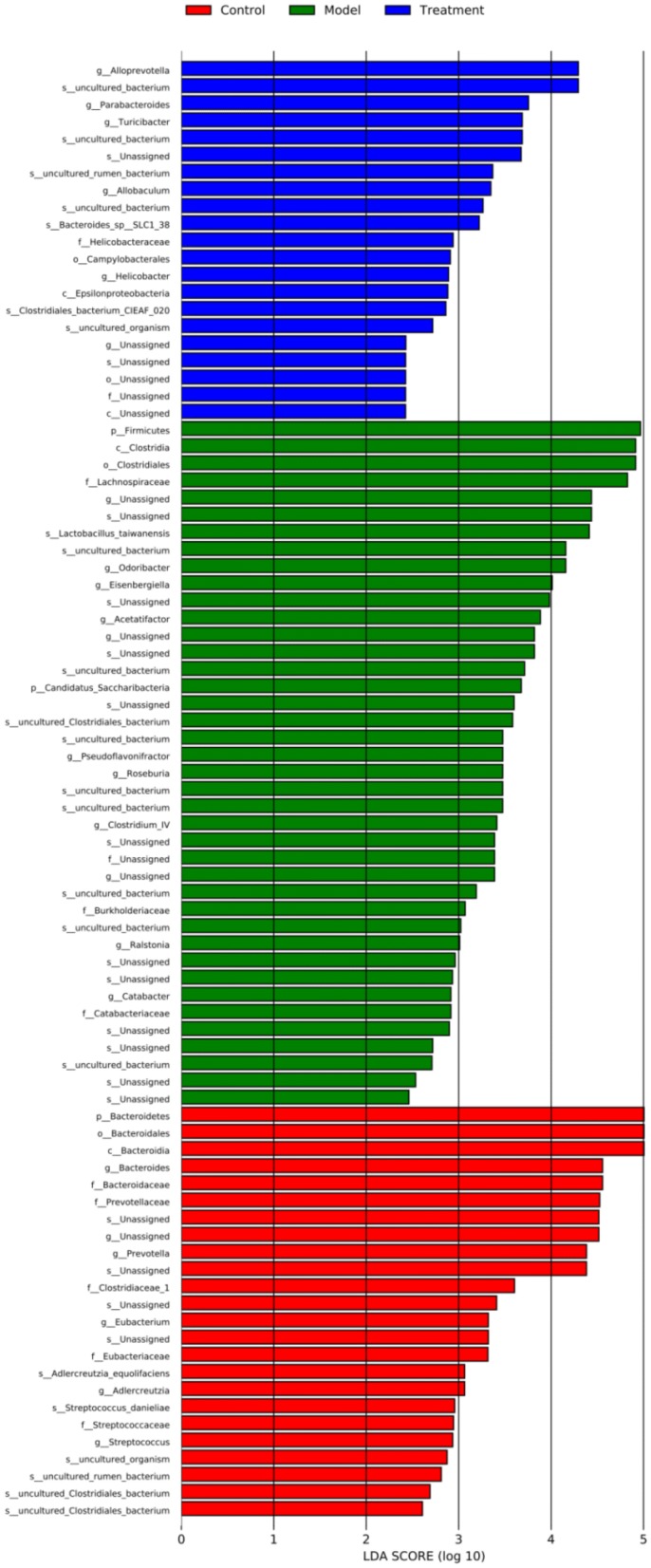
LDA scores for the differentially abundant bacterial taxa among the three groups. LDA scores > 2.0 are shown. Treatment group indicates acupoints combination treatment group

**Fig. 9:**
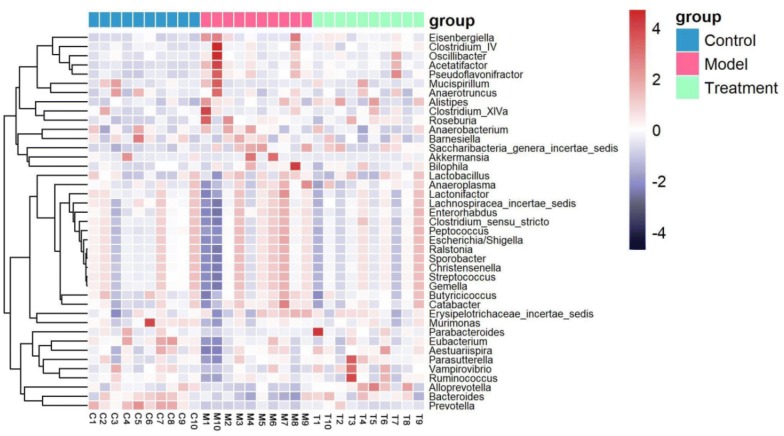
Heat-map of bacterial taxa of mice in the three groups. Treatment group indicates acupoints combination treatment group

**Fig. 10:**
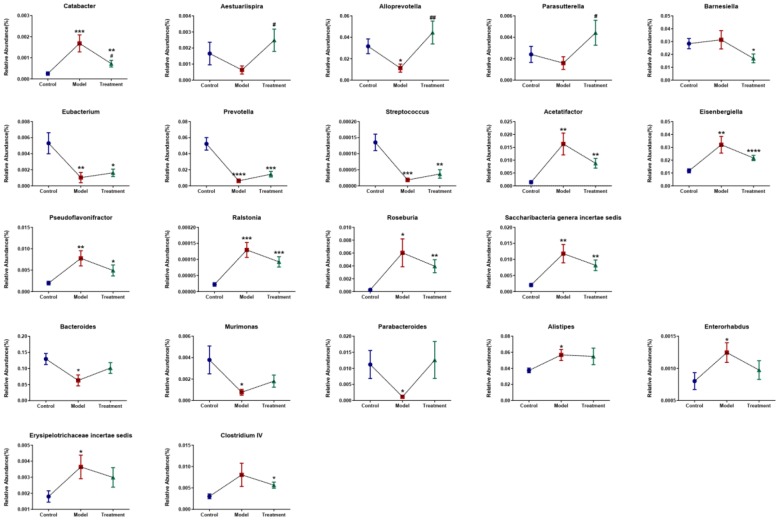
Comparison of the relative abundance (%) of bacterial taxa at the genus level. The significant differences of bacterial taxa at the genus level among the three groups were identified using one-way ANOVA. *Indicates model or treatment (acupoints combination) group versus control group. *p < 0.05; **p < 0.01; ***p < 0.001; ****p < 0.0001. ^#^Indicates treatment (acupoints combination) group versus model group. ^#^*p* < 0.05; ^##^*p* < 0.01

Since acupuncture is a comprehensive and holistic treatment to adjust the systemic status of living organism [57]. Acupuncture may not directly target tumor to postpone tumor’s development. Therefore, we focused on cells and factors related to immune system, and gut microbiota to further determine the effect of acupuncture on tumor burdened mice, as immune response and gut microbiota have been suggested to have a close relation to both acupuncture and tumor [58–61]. For a clear experimental observation, we used mice of needling acupoints combination treatment group to perform both tests of immune cells and gut microbiota after treatment period of phase III. First, we used flow cytometry to detect immune cells in the spleen and subaxillary lymph nodes of mice, including T cells and NK cells respectively. The results showed that there was a significant increase of NK cells in the model group compared to the control group (Fig. 3). As NK cells are the important defender to eliminate tumor cells, the increased secretion of NK cells reflected that the immune system was active against tumor. However, the contents of NK cells of mice in the acupoints combination treatment group were significantly lower than that of the model group. Given the previous results observed from in vivo imaging and weight of tumor burdened mice (Fig. 2), the results of NK cells of treatment group may indicate that the immune system has not yet produced as same amount of NK cells as model group to fight tumor, since tumor grow slowly in acupuncture treatment group (Fig. 3). In addition, T cells in the spleen are divided into toxic and helper T cells. Toxic T cells have immunosuppressive effects, and increased levels of CTLs indicate susceptibility to cancer or autoimmune disease. By contrast, helper T cells release a variety of cytokines that participate in anti-tumor effects. Our results showed that compared to the control group, the number of helper T cells was significantly lower in the model group, but the number of toxic T cells was significantly higher in the model group (Fig. 3). The results have revealed that the state of these immune cells has been changed significantly with the development of tumor, in particular the increased expression of toxic T cells indicated the influence of tumor on immune system of mice. Moreover, the number of both helper and toxic cells in the acupuncture treatment group was significantly different compared to the control and model group, respectively. The expression of helper T cells in the acupuncture treatment group was lower than that in the control group, but higher than that in the model group. By contrast, the secretion of toxic T cells in the acupuncture treatment group was higher than that in the control group, but lower than that in the model group. These results indicated again the changes of immune cells were postponed on tumor burdened mice by using acupuncture treatment. In addition, the levels of IL-6, TNF-α, and IFN-γ in the serum and spleen were analyzed by ELISA. The results demonstrated highly similar tendency compared to the results obtained from the tests of immune cells (Fig. 3), which also indicated acupuncture treatment may delay the development of tumor. Next, we concentrated on gut microbiota of tumor burdened mice to further evaluate the effect of acupuncture treatment. Figure 4a shows that the total intestinal flora differed among the different groups of mice. In addition, the number of observed species increased with the amount of sequencing data rising in the rarefaction curve. When the amount of sequencing data reached a certain value, the rarefaction curve showed a relatively steady situation, indicating that the diversity index slowly increased while the platform had been achieved (Fig. 4b). Figure 5 shows the dominant populations and their relative abundance in the three groups at the phylum level. In the three groups, *Bacteroidetes* and *Firmicutes* were the bacteria with the highest relative abundance. Their levels in the model and acupuncture treatment groups were significantly different from those in the control group. In addition, the content of the two bacteria in the acupuncture treatment group was also significantly different from that in the model group, and it was closer to that in the control group. Similar results were found with *Candidatus Saccharibacteria.* To further study the differences in intestinal microorganisms in the three groups, unsupervised PCoA was applied to the OTU data at the phylum level to visualize the variations among the different groups. Figure 6 shows the PCoA results in the form of a score plot. The results showed that the variance in OTU data of mice due to the two principal components (Axis 1 and Axis 2) accounted for 45.55% and 17.53% of total variance, respectively. The results also showed three clusters among the groups. In addition, the acupuncture treatment group was closer to the control group than to the model group on the PCoA score plot. Next, to identify specialized communities in samples, we used the LEfSe tool on the I-Sanger platform. LEfSe enables comparisons between multiple groups, to identify bacterial species with significant differences in abundance between groups. Figure 7 is a cladogram diagram of bacterial abundance in the three groups. The circle diameter represents the abundance of the species. The circles that radiate from the inside to the outside represent the classification level from phylum to species (phylum–class–order–family–genus–species). Each small circle at a different classification level represents a classification at that level. The diameter of the small circle is proportional to the relative abundance. The area of the fan drawn from the inside out is the level of species annotation. Species with no significant differences are uniformly colored yellow. Gut microbiota are colored differently, the red nodes represent the microbial groups that play an important role in the control group, the green nodes represent the microbial groups that play an important role in the model group, and the blue nodes represent the microbial groups that play an important role in the acupuncture treatment group. Figure 8 shows the LDA scores of the differentially abundant taxa in the three groups. The length of bar chart represents the contribution of specific bacteria within the group. Next, we focused on the differences among the three groups at the genus level. Figure 9 is a heat map of 41 genera of bacteria in the groups, differences among the groups can be seen, but they were not significant. We used ANOVA to identify 21 bacteria with significant differences among the three groups at the genus level (Fig. 10). Compared to the model group, the bacterial abundance in the acupuncture treatment group was closer to that in the control group. Similar results were found in 20 other genus level bacteria (Additional file 4: Fig. S2). Almost all the results in gut microbiota study have shown that the significantly different level of bacteria between control and model group, however, the expression of bacteria of acupuncture treatment group was in between of control and model group. These results demonstrated that acupuncture treatment adjusted the expression of specific bacteria, and leaded to the relatively slow changes of bacteria compared to the model group.

## Discussion

According to the traditional Chinese medicine theory, one’s health depends on a dynamic and holistic balance between one’s physiological state and the surrounding environment [62, 63]. Illness is viewed as a disruption of the body’s dynamic and holistic balance. Acupoints are the special points of the body where viscera, meridians, “Qi” and “Xue” exist on the body surface. Needles are used to stimulate acupoints in order to trigger a comprehensive and systemic adjustment to balance the disruption on sick body to recover from illness. In acupuncture therapy, the choice of acupoints is a key factor to ensure the curative effects of acupuncture and moxibustion [64, 65]. Yang deficiency is the main pathogenesis of cancer based on the principle of traditional Chinese medicine. Therefore, we selected the acupoints with the closest relationship to Yang qi and vitality (Shenshu [BL23], Baihui [DU20], and Zusanli [ST36]) [66–69]. Our results show that stimulating the combination of these acupoints can obtain the best effects on delaying of tumor growth. This results are in accordance with previous research [46, 70, 71]. It also indicates that needling different acupoints together may lead to a synergistic effect which postponed strongly the tumor growth, however, the potentially synergistic effect need to be further studied to evaluate the detailed differences between stimulating specific acupoints and acupoints combination. To study the body changes of mice inoculated tumor by needling acupoints, we first focused on the expression of specific immune cells and inflammatory cytokines.

The results showed that compared to the model group, the expression level of all those cells and cytokines of mice received acupuncture treatment were closer to that of the control group (Fig. 3). As tumor development has been associated with immunomodulatory response of body [46]. Our results also suggest a slow development trend of tumor from a view of cellular and molecular levels. Cellular immunity is the main immune response to the body’s own immune mechanism. T cells and NK cells are involved in the immune process [70, 71]. The immune system is weak when body suffers tumor, in part due to the imbalance in the proportion of immune cells and related cytokines. Wu et al. [72] explored the role of acupuncture in regulating cellular immunity, showing it can increase the levels of CD3+ and CD4+ T cells, and decrease the levels of cytotoxic CD8+ T cells, so that the CD4+/CD8+ ratio is close to normal range, improving the body’s immune response [73]. Pei [74] found that acupuncture can increase the IFN-γ expression of serum of patients who suffered non-small lung cancer. Zhang et al. [75] found that using the combination between cisplatin and acupuncture can significantly reduced TNF-α levels of patients who suffered advanced lung cancer, in turn, possibly improving the immune function and clinical efficacy of patients. These studies all support the results of this research.

Next, we concentrated on the changes of intestinal flora on tumor burdened mice, some cancers and cancer-associated illnesses reportedly have abnormal intestinal flora [25, 26, 28, 75, 76]. In this study, it was observed that the tumor burdened mice also developed significant changes of intestinal flora. At the phylum level, the significant changes of model group were mainly observed in *Bacteroidetes, Firmicutes* and *Candidatus Saccharibacteria* compared to the control group (Fig. 5). Acupuncture treatment works on delaying the decrease of *Bacteroidetes* and the increase of *Firmicutes* and *Candidatus Saccharibacteria*. In addition, a significant imbalance of intestinal microflora was observed at the genus level, leading to an increase of *Catabacter, Acetatifactor, Pseudoflavonifractor, Ralstonia, Roseburia, Saccharibacteria genera incertae sedis, Alistipes, Erysipelotrichaceae incertae sedis,* and *Clostridium IV*; and a decrease of *Aestuariispira, Alloprevotella, Parasutterella, Eubacterum, Prevotella, Streptococcus, Bacteroides, Murimonas,* and *Parabacteroides* of model group compared to the expression of those bacteria of control group. However, acupuncture can regulate these changes of intestinal flora. Compared to the model group, the bacterial abundance at both the phylum and genus levels of the acupuncture treatment group was closer to that of the control group. This also indicates the effect of acupuncture treatment on tumor burdened mice can be addressed on delaying disease development. It is worth noting that patients with osteosarcoma often have diarrhea, constipation, and other gastrointestinal conditions [77]. In fact, patients with other cancer-related diseases also often have severe diarrhea and constipation [78]. At present, the treatment of cancer patients usually takes the form of high-dose chemotherapy, but it often leads to strong gastrointestinal reactions. Various treatments for other cancers often also cause diarrhea [79]. These reports indicate that cancer is related to the disorder of intestinal flora, which is targeted by current western medicine treatments. This study found that acupuncture can maintain the body’s immunity, regulate the secretion of inflammatory factors, regulate gut microbiota, and interfere with tumor growth. Therefore, acupuncture as a complementary and holistic treatment may have a big potential to promote cancer therapy and management.

## Conclusion

In this research, we found that acupuncture can delay tumor growth in mice burdened osteosarcoma tumor, and postpone the changes of immunomodulatory response and gut microbes in those mice, suggesting that acupuncture may provide a holistic and systematic treatment for cancer. Our findings encourage new and extensive research into the effects of acupuncture on the composition of the gut microbiome that lead to the development of cancer. Because of the close relationship between the immune system and intestinal flora [59–61], further research should combine immunological and microbiome data to further assess the molecular effects of acupuncture on cancer. In addition, the link among immunomodulatory response, microbiota and acupuncture treatment should be further investigated to study details and mechanism of changes of immune cells and bacteria by needling acupoints. In the long term, our work is important for understanding the comprehensive systemic role of acupuncture in the treatment of cancer.

## Supplementary information

**Additional file 1.** Original data 1.

**Additional file 2.** Original data 2.

**Additional file 3: Figure S1.** The tumor volume and weight obtained by the treatment of four different treatment groups.

**Additional file 4: Figure S2.** Comparison of the relative abundance (%) of bacterial taxa at the genus level.

## Data Availability

The datasets used in this study are available from the corresponding author upon reasonable request.
