# Supplementary material for: The effect of acupuncture on tumor growth and gut microbiota in mice inoculated with osteosarcoma cells
Source: Chin Med. 2020 Apr 7;15:33. doi: 10.1186/s13020-020-00315-z (PMC7140491; doi:10.1186/s13020-020-00315-z)
Supplement: Supplementary file 3 — Additional file 3: Figure S1. The tumor volume and weight obtained by the treatment of four different treatment groups. [file 13020_2020_315_MOESM3_ESM.docx]

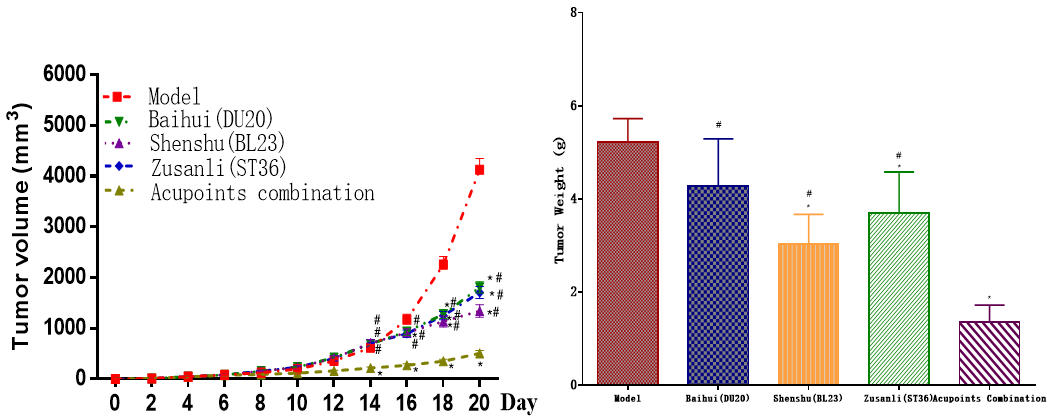


**Figure S1. The tumor volume and weight obtained by the treatment of four different treatment groups.** Tumor volumes of mice in the model and different treatment groups. Schematic diagram of tumor weight of mice in the model and the four different treatment groups after the acupuncture treatment procedure.*indicates the four different treatment groups versus model group, *p* <0.05 (two-tailed, unpaired Student’s *t*-test). # indicates the other three treatment groups versus acupoints combination treatment group, *p* <0.05 (two-tailed, unpaired Student’s *t*-test).
