# Supplementary material for: The effect of acupuncture on tumor growth and gut microbiota in mice inoculated with osteosarcoma cells
Source: Chin Med. 2020 Apr 7;15:33. doi: 10.1186/s13020-020-00315-z (PMC7140491; doi:10.1186/s13020-020-00315-z)
Supplement: Supplementary file 4 — Additional file 4: Figure S2. Comparison of the relative abundance (%) of bacterial taxa at the genus level. [file 13020_2020_315_MOESM4_ESM.docx]

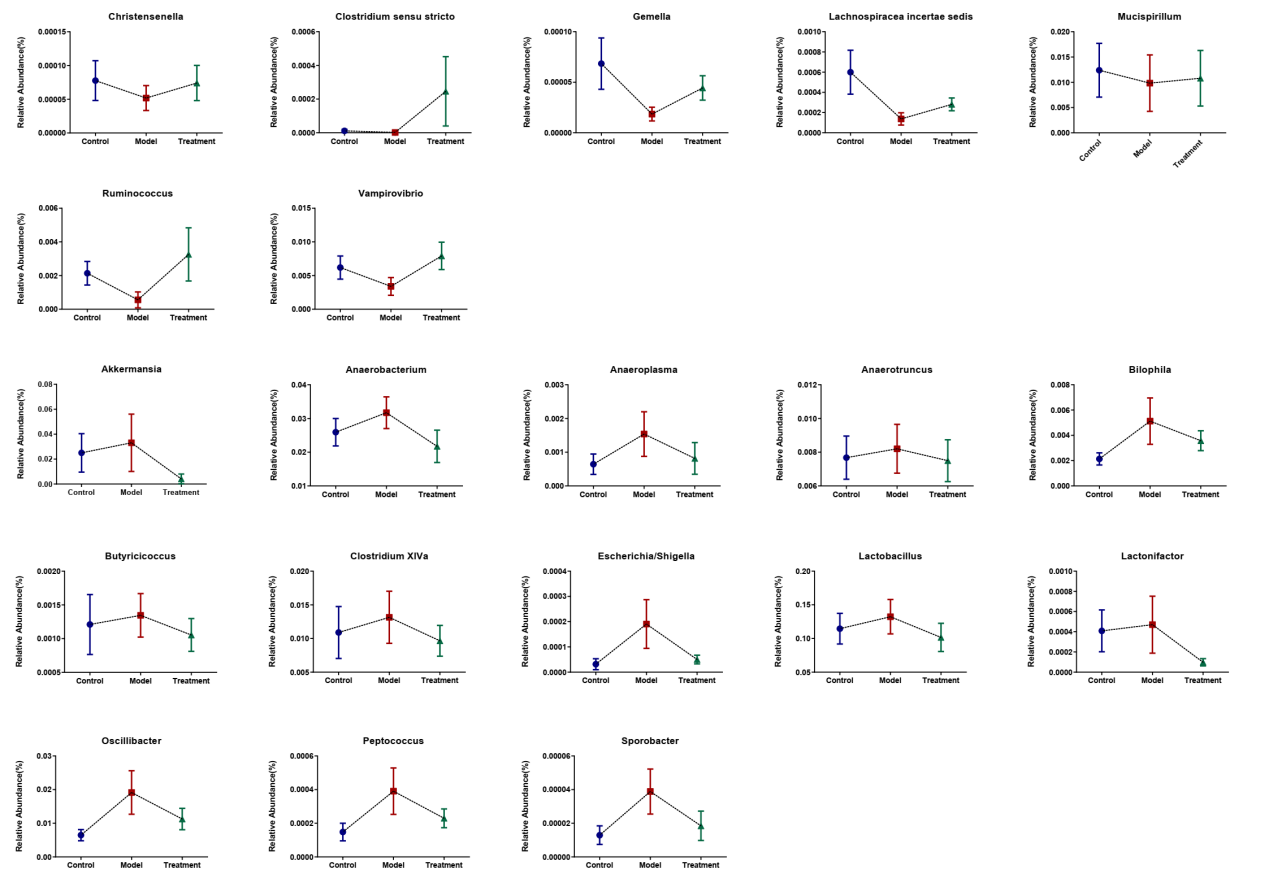


**Figure S2. Comparison of the relative abundance (%) of bacterial taxa at the genus level.** Treatment group indicates acupoints combination treatment group.
